# Stability of Vitamin A, Iron and Zinc in Fortified Rice during Storage and Its Impact on Future National Standards and Programs—Case Study in Cambodia

**DOI:** 10.3390/nu8010051

**Published:** 2016-01-16

**Authors:** Khov Kuong, Arnaud Laillou, Chantum Chea, Chhoun Chamnan, Jacques Berger, Frank T. Wieringa

**Affiliations:** 1 DFPTQ, Fisheries Administration, MAFF, 186 Preah Norodom Boulevard, 12000 Phnom Penh, Cambodia; kuong.kh@gmail.com (K.K.); chhounchamnan@gmail.com (C.C.); 2 UNICEF, Maternal, Newborn and Child Health and Nutrition section, No. 11 street 75, 12202 Phnom Penh, Cambodia; 3 National Sub-Committee of Food Fortification, Ministry of Planning, 386 Monivong Blvd, 12000 Phnom Penh, Cambodia; chantumch@gmail.com; 4 Institute of Research for Development (IRD), UMR Nutripass IRD-UM2-UM1, 3400 Montpellier, France; jacques.berger@ird.fr (J.B.) franck.wieringa@ird.fr (F.T.W.)

**Keywords:** fortification, rice, vitamin A, iron, zinc, stability, Cambodia

## Abstract

Fortified rice holds great potential for bringing essential micronutrients to a large part of the world population. The present study quantified the losses of three different micronutrients (vitamin A, iron, zinc) in fortified rice that were produced using three different techniques (hot extrusion, cold extrusion, and coating) and stored at two different environments (25 ± 5 °C at a humidity of 60% and 40 ± 5 °C at a humidity of 75%) for up to one year. Fortified rice premix from the different techniques was mixed with normal rice in a 1:100 ratio. Each sample was analyzed in triplicate. The study confirmed the high stability of iron and zinc during storage while the retention of vitamin A was significantly affected by storage and the type of techniques used to make rice premix. Losses for iron and zinc were typically <10% for any type of rice premix. After 12 months at mild conditions (25 °C and humidity of 60%), losses for vitamin A ranged from 20% for cold extrusion, 30% for hot extruded rice 77% for coated rice premix. At higher temperatures and humidity, losses of vitamin A were 40%–50% for extruded premix and 93% for coated premix after 6 months. We conclude that storage does lead to a major loss of vitamin A and question whether rice is a suitable food vehicle to fortify with vitamin A. For Cambodia, fortification of rice with iron and zinc could be an effective strategy to improve the micronutrient status of the population if no other food vehicles are available.

## 1. Introduction

The elimination of micronutrient deficiencies in vulnerable groups is a public health priority for many countries, regardless of economic development. The Copenhagen Consensus 2012 has put combating nutritional deficiencies as one of the most important strategy to improve health [1], especially in low- and middle-income countries where the burden of micronutrient malnutrition is enormous. It has been estimated for example that Cambodia will lose $150 to $200 US million a year if deficiencies of iron, iodine, vitamin A and zinc persist [2]. Many countries have taken steps to reduce micronutrient deficiencies through different interventions [3]. Direct supplementation has been recognized to be the fastest method to reduce micronutrient deficiencies, and many supplementation programs have been put in place, such as vitamin A supplementation for young children and iron plus folate supplements for pregnant women. However, these programs depend heavily on compliant behavior, consistent funding from the government or international donors, and national healthcare distribution channels that may or may not be equally accessible for the entire population. Dietary diversification programs are not dependent on external funding or the health care system, but often struggle to change long-established behaviors and, therefore, quick impacts are difficult to achieve over a short period of time. Food fortification is often seen as the most cost-efficient intervention to address micronutrient deficiencies. At the Copenhagen Consensus in 2008, fortification of staple foods with micronutrients (*i.e.*, vitamins and minerals) was ranked among the top three international development priorities [4].

Indeed, fortification of selected food items can be an excellent tool to improve the status of specific micronutrients. For example, in Vietnam, the prevalence of anemia decreased and iron status improved among women of reproductive age consuming fish sauce fortified with iron [5]. Cambodia has difficulties finding an effective solution to ensure that its population has an adequate level of micronutrients. The recent 2014 Cambodian Demographic Health survey (DHS) showed that in comparison to the two earlier national demographic health surveys [6,7], the prevalence of anemia among children and women has not reduced significantly, and remains high at 55.5% and 45.4%, respectively. The recent data on micronutrient status from the 2014 CDHS shows the need for new programs. Though, over the last 4 years (2010–2014), the government of Cambodia has been distributing iron-folate supplements to women of reproductive age, and micronutrient powders to children 6–24 months [8], almost 40% of the women had a marginal iron store and 20%–28.6% of the children aged 6–24 months have been diagnosed with iron-deficient-anaemia [6]. Prevalence of zinc deficiency was very high also, with over half of the mothers and children having low zinc concentrations (Wieringa, personal communication).

Fortification of staple foods such as rice could be a promising strategy for Cambodia, where approximately 70% of the daily energy intake comes from rice [9]. Decisions about which nutrients to add and in which amounts must be guided by the nutritional needs of the population, the rice consumption profile of the target groups, other programs already in place and their coverage. Assessing the bioavailability and stability of the micronutrients added to those staple foods is also essential to be able to estimate the potential impact a fortification program can have. Any overage to be added to compensate losses during storage and cooking will have a direct impact on the sustainability of a program due to its additional cost. Recently, Wieringa *et al.* [10] showed that cooking fortified rice (coated, cold and hot extruded rice) in excess water without soaking destroys almost 80% of the added vitamin A as retinyl palmitate. This is important as vitamin A is one of the most expensive vitamins in premix and the current overage of 50% might not be sufficient [10].

The purpose of the present study was, therefore, to determine the influence of time (“shelf life”) and temperature/humidity on the stability of vitamin A, iron and zinc. We also assessed the potential standard in order to start a rice fortification program in Cambodia according to the consumption of rice, the losses of the different nutrients, the other known interventions and national prevalence of micronutrient deficiencies.

## 2. Experimental Section

### 2.1. Type of Rice Premix

Fortified rice suitable for human consumption is normally made by mixing artificial fortified rice kernels, also referred to as rice premix, with normal rice kernels. Rice premix is made by addition of a vitamin-mineral premix to either rice flour (hot- and cold-extrusion methodologies) or as a coat over normal rice kernels (coating methodology).

Three different types of production methods for making rice premix were tested. Rice premix was requested from six producers of fortified rice using different methods. Three different methods of producing fortified rice were tested: (1) coating of rice kernels with a mixture of premix (vitamins and minerals) and waxes sprayed on normal rice kernels; (2) cold extrusion of a mixture of rice flour, premix (vitamins and minerals), and a binding agent, such as alginate; (3) hot extrusion of a mixture of rice flour and premix (vitamins and minerals). Differences between cold and hot extrusion include the temperature (cold extrusion, <70 °C; hot extrusion, >70 °C), use of steam (hot extrusion), and the inclusion of a binding agent (necessary for cold extrusion, whereas gelatinized starches from the rice provide the binding agent for hot extrusion) [11]. For each method, two reputed manufacturers were invited, hence there were six manufactures in total. All rice premixes were made by using the same vitamin-mineral premix, provided to the manufacturers by the Global Alliance for Improved Nutrition (GAIN) premix facility (see below).

All producers were provided with the same premix containing 458 mg of ferric pyrophosphate, 7 mg of folic acid, 50 mg of vitamin B_12_ (0.1% WS), 100 mg of retinyl palmitate, and 183 mg of zinc oxide per g premix. The mixing ratio of rice premix to normal rice kernels was 1% (1:100 ratio). The anticipated losses of the micronutrients during production of the fortified kernels were unknown but arbitrarily set at 10% for zinc oxide and ferric pyrophosphate and 50% for vitamin A (Table 1) [11]. As the rice premix was mixed in a 1:100 ratio with normal rice, the amount of micronutrients in 1 g of rice premix is the same as the target amount of micronutrients in 100 g of prepared rice, assuming limited amount left of the three micronutrients in normal rice once cooked (less than 0.4mg of zinc and less than 0.1mg for iron and no vitamin A in 100g of white rice (http://nutritiondata.self.com/facts/cereal-grains-and-pasta/5722/2#ixzz3wG64QkYo). Fortification levels were chosen to meet ~30% of the recommended nutrient intake for a 19- to 50-year-old, non-pregnant, non-lactating woman corresponding to 600 μg of retinol equivalents, 10 mg of iron and 14 mg of zinc (Table 1).

**Table 1:** Target levels for fortified rice as kernel and for 100 g of uncooked rice.

Micronutrient	Target Value in 1 g of Fortified Kernel (mg)	Anticipated losses (%)	Mean Concentration (mg ± SD) as Determined in 100 g Uncooked Rice
Iron	3.3	10	3.31 (± 0.30)
Zinc	4.4	10	5.3 (± 0.34)
Vitamin A	0.225	50	0.157 (± 0.024)

### 2.2. Mixing and Sampling

Rice premix samples from the six manufacturers were sent to a laboratory in Denmark (Eurofins Steins Laboratorium, Vejen, Denmark) that blended all rice premix (coated, cold-extruded, and hot-extruded) with normal rice. To make fortified rice, normal blending ratios range from 1:200 to 1:50. In the present study, the rice premix from the six manufacturers was mixed with normal rice to obtain fortified rice premix to normal ratio in a 1:99 ratio [10]. The normal rice used was Thai Jasmine rice (Super Lucky Elephant, San Francisco, CA, USA). All analyses were carried out on 200 g portions of fortified rice (*i.e.*, 2 g of rice premix with 198 g of regular rice). The same regular rice was used across all tests. Rice premix and normal rice were mixed for 5 min using a TURBULA^®^ Powder Blender (Glen Mills, Inc., Clifton, NJ, USA) in order to guarantee homogenous mixing of the samples.

### 2.3. Storage Condition

Micronutrient concentrations were analyzed at Eurofins Steins Laboratorium (Vejen, Denmark) under the conditions usually encountered in developing countries (25 ± 5 °C at a humidity of 60% and 40 ± 5 °C at a humidity of 75%). The test aimed to mimic the conditions in several retailers or warehouse and kitchens. The fortified rice were stored within their traditional bags. Micronutrients contents of the three different batches (coated, hot-extruded, cold-extruded) were analyzed at baseline, three, six and 12 months later (respectively called T0, T90, T180, T360) for 25 ± 5 °C at a humidity of 60%. At a higher temperature and humidity (40 ± 5 °C at a humidity of 75%), another timeline was set as we expected a more rapid degradation of the vitamin A. Therefore we analysed the batches at baseline, one, three and six months later (respectively called T0, T30, T60, T180).

### 2.4. Determination of Micronutrient Content

All micronutrient contents were outsourced and performed by Eurofins Steins Laboratorium (Vejen, Denmark). Vitamin A content of the uncooked rice was determined using reverse-phase HPLC, with detection by an ultraviolet/diode array detector at 325 nm [12]. From the homogenized samples, 15 g of rice was mixed with 500 mg of takadiastase and 25 mL of demineralized water. Samples were shaken for 30 min at 25 °C. Vitamin A was saponified using ethanoic potassium hydroxide solution and extracted three times with hexane/ethylacetate (85:15 *v*/*v*). Iron and zinc content of the rice was determined using inductive coupled plasma-optic emission spectrometry [13]. A small amount of sample was weighed into a Teflon tube, and nitric acid and hydrogen peroxide was added. The tube was then sealed and placed in a microwave oven to dissolve the iron and zinc from the sample before analysis.

### 2.5. Potential Contribution to Cambodian Daily Diet

Recently, a technical working group has agreed on potential level for rice fortification [14]. According to a national rice consumption of approximately 250 g/day, the recommended levels at moment of consumption are 7 mg of iron (preferred form: micronized ferric pyrophosphate), 0.15 mg of vitamin A (as retinol palmitate) and 6 mg of zinc (as zinc oxide) per 100 g of rice. These targets do not take into consideration the losses during the storage of the fortified rice between production and consumption. In Cambodia, we have estimated this time at three months at 40 °C and 75% humidity. Therefore, we have estimated the minimum level of fortification required for Cambodia due to storage (actual findings) and cooking losses [10].

### 2.6. Statistical Analysis

For each micronutrient concentration analysis (*i.e.*, each type of rice premix uncooked in triplicate), a coefficient of variation (CV) of <20% was considered acceptable. Data with a CV > 20% were checked for the high variations, and, if inexplicable, excluded from statistical analysis. Retention values are given as a percentage of the amount of micronutrients present in the uncooked fortified rice per dry matter at T0 compared to the amount of micronutrient present in the uncooked rice per dry matter after each time period at the given conditions.

Differences between each uncooked fortified rice at T0, T90, T180 and T360 were calculated using analysis of variance (ANOVA), with a *p* value < 0.05 being considered statistically significant. The interaction between production method and time of storage was considered significant if *p* < 0.05, which was the case for all micronutrients. *Post-hoc* tests between the different fortified rice and between the times of the storage were carried out using Bonferroni correction for multiple comparisons. Data were analyzed using SPSS Statistics (Release 19.0.0, IBM, Armonk, NY, USA).

## 3. Results

### 3.1. Vitamin A

Under the tested storage conditions and at each time point, retentions of retinyl palmitate (Table 2) were always significantly higher in extruded fortified rice (hot or cold) than in coated fortified rice (*p* < 0.05).

At no time point was there a significant difference between hot- and cold-extruded rice. After three months of storage at 25 °C, the vitamin A concentration remained similar to baseline for both extrusion methodologies, while more than 20% of the vitamin A was lost in the coated rice. However, at higher temperature and humidity (40 °C/75%), all types of fortified rice had significant losses at three months, ranging from 22% (hot extrusion) to 82% (coating) of the initial concentration of retinyl palmitate. After six months of storage, only the hot-extruded rice stored at 25 °C was still not significantly different from baseline, whereas cold-extruded rice had lost 16% and coated rice even 56% of its vitamin A content. After 6 months at higher temperature and humidity, losses increased to 22%, 35% and 88%, respectively, for hot-extruded, cold-extruded and coated rice. Losses after one year ranged from 21% (hot-extruded, 25 °C) to 93% (coated rice, 40 °C).

**Table 2:** Retention of retinyl palmitate over time, as percentage from retinyl palmitate concentration in uncooked fortified rice prior to storage (T0).

	**25 °C/60% Humidity**			
***Type***	**T0**	**T90**	**T180**	**T360**
Hot extrusion	100	103.1 (± 5.1) ^c,5^	90.6 (± 7.0) ^c^	78.9 (± 12.9) ^c,1,3^
Cold extrusion	100	94.6 (± 12.1) ^c,5^	83.4 (± 5.8) ^c,1^	70.1 (± 8.0) ^c,1,3^
Coated	100	77.5 (± 7.7) ^a,b,1,4,5^	43.7 (± 14.2) ^a,b,1,3,5^	23.1 (± 15.8) ^a,b,1,3,4^
	**40 °C/75% Humidity**			
***Type***	**T0**	**T30**	**T90**	**T180**
Hot extrusion	100	78.7 (± 5.5) ^c,1,4^	78.0 (± 13.7) ^c,1,4^	51.5 (± 14.2) ^c,1,2,3^
Cold extrusion	100	80.1 (± 2.3) ^c,1,3,4^	64.7 (± 7.0) ^c,1,2,4^	39.3 (± 5.7) ^c,1,2,3^
Coated	100	40.6 (± 15.2) ^a,b,1,3,4^	17.6 (± 14.1) ^a,b,1,2^	6.9 (± 7.8) ^a,b,1,2^

Note: All values are means ± SD. ^1^ significantly different from T0; ^2^ significantly different from T30; ^3^ significantly different from T90; ^4^ significantly different from T180; ^5^ significantly different from T360; ^a^ significantly different from Hot extrusion; ^b^ significantly different from Cold extrusion and ^c^ significantly different from Coated.

### 3.2. Iron and Zinc

Over time and regardless of the temperature and humidity conditions, iron (Table 3) and zinc (Table 4) concentrations were, in general, similar to baseline, with no significant differences observed except for zinc concentrations at T30 (40 °C/75% humidity) in hot-extruded and coated rice, and for iron at T360 (25 °C/60% humidity) in hot-extruded rice. Retention was typical high at 90%–100%

**Table 3:** Retention of iron over time, as percentage from iron concentration in uncooked fortified rice prior to storage (T0).

	**25 °C/60% Humidity**			
***Type***	**T0**	**T90**	**T180**	**T360**
Hot extrusion	100	94.7 (±2.2) ^b^	98.0 (±3.5) ^b^	89.9 (±6.7) ^1^
Cold extrusion	100	109.5 (±11.4) ^a^	109.9 (±5.7) ^a,c^	100.5 (±10.3)
Coated	100	101.1 (±9.3)	92.9 (±5.0) ^b^	91.2 (±7.4)
	**40 °C/75% Humidity**			
***Type***	**T0**	**T30**	**T90**	**T180**
Hot extrusion	100	82.1 (±27.1)	97.6 (±5.7)	89.4 (±2.0) ^b^
Cold extrusion	100	99.4 (±6.2)	106.3 (±8.2)	97.7 (±6.8) ^a^
Coated	100	100.3 (±13.6)	99.4 (±6.4)	91.1 (±4.2)

Note: All values are means ± SD. ^1^ significantly different from T0; ^a^ significantly different from Hot extrusion; ^b^ significantly different from Cold extrusion and ^c^ significantly different from Coated.

**Table 4:** Retention of zinc overtime, as percentage from zinc concentration in uncooked fortified rice prior to storage (T0).

	**25 °C/60% Humidity**			
***Type***	**T0**	**T90**	**T180**	**T360**
Hot extrusion	100	99.5 (± 2.8)	94.5 (± 2.8)	100.8 (± 8.0)
Cold extrusion	100	103.1 (± 7.0) ^4^	93.7 (± 5.4) ^3^	101.8 (± 3.9)
Coated	100	102.3 (± 8.3) ^4^	91.0 (± 3.5) ^3^	96.5 (± 6.0)
	**40 °C/75% Humidity**			
***Type***	**T0**	**T30**	**T90**	**T180**
Hot extrusion	100	87.6 (± 6.2) ^b,1,3,4^	98.5 (± 2.3) ^2^	96.3 (± 2.5) ^2^
Cold extrusion	100	99.5 (± 5.8) ^a,c^	100.4 (± 4.4)	101.9 (± 8.9)
Coated	100	87.9 (± 6.8) ^b,1,3,4^	99.8 (± 3.8) ^2^	96.8 (± 4.3) ^2^

Note: All values are means ± SD. ^1^ significantly different from T0; ^2^ significantly different from T30; ^3^ significantly different from T90; ^4^ significantly different from T180; ^a^ significantly different from Hot Extrusion; ^b^ significantly different from Cold Extrusion and ^c^ significantly different from Coating.

### 3.3. Potential Contribution to Cambodian Daily Diet

Using these losses during typical storage conditions in Cambodia (three months at 40 °C and 75% humidity), and the reported losses during cooking [10], we calculated specific target fortification levels for iron and zinc based on the current recommendations [14] and we found that rice should be fortified at the level of 1.10–16.67 mg of retinyl palmitate, 7.0–8.24 mg for iron and 6.32–6.67 mg of zinc per 100 g of rice depending on the technology (Table 5).

**Table 5:** Potential standard recommendation for fortified rice in Cambodia for retinyl palmitate, iron and zinc.

Type of Fortification	Micronutrient	Recommended Technical Standard (mg/100 g of Fortified Rice)	Loss during Storage (Three Months)	Recommended Quantity to Overcome Storage Losses	Maximum Loss during Cooking (%)	Potential Cambodia Standard (mg/100 g of Fortified Rice)
***Coated***	Iron	7	-	7	11%	7.74
	Zinc	6	-	6	10%	6.60
	Vitamin A	0.15	82%	0.27	95%	0.53
***Hot Extrusion***	Iron	7	-	7	15%	8.04
	Zinc	6	-	6	10%	6.57
	Vitamin A	0.15	22%	0.18	86%	0.34
***Cold extrusion***	Iron	7	-	7	0%	7.00
	Zinc	6	-	6	5%	6.29
	Vitamin A	0.15	35%	0.2	79%	0.32

Assumptions made: ^1^ Cambodian are boiling their rice; ^2^ fortified rice is not stored more than three months at 40 °C and 75% of humidity after being fortified; ^3^ the Cambodian daily rice consumption is between 150 and 300 g; ^4^ “-”: almost no losses.

## 4. Discussion

This study complements the recently published article on the stability and retention of micronutrients in fortified rice prepared using different cooking methods [10]. For iron and zinc, our study confirms the high stability of those two compounds even during storage at high temperatures (40 °C) and high humidity (75%) for 12 months. Both compounds, micronized iron pyrophosphate and zinc oxide, have no significant losses over months of storage. In contrast, vitamin A as retinyl palmitate was significantly affected during storage, with losses up to 90% at the highest temperature and humidity.

In the current study, the amount of vitamin A in the rice premix at the start of the study was close to the target value of 0.15 mg per 100 g of rice, as recently proposed [14]. In coated fortified rice, vitamin A concentrations rapidly diminished, with over 50% of the vitamin A being lost within six months in the mildest condition tested. At higher humidity and temperature (75%, 40 °C), 80% of the vitamin A was lost within six months, and almost 94% was lost after one year. Such high losses cannot be corrected by adding higher overages, as the fortification level at T0 would be unacceptably high. Especially children are at risk for vitamin A toxicity after long-term high vitamin A intakes, with toxicity reported at 1500 IU of vitamin A/kg body weight (5000 μg of retinol equivalents/kg body weight) [15]. Hence, the results of the present study suggest that the current techniques for making fortified rice through coating are inadequate for vitamin A and the addition of vitamin A to the premix of coated rice is inefficient in regard to the added cost of the overage.

The retention of vitamin A in the other two types of extruded rice was much better with 0%–5% of the vitamin A lost after three months, 10%–16% after six months and 21% and 30% after one year at mild conditions, suggesting a slow but steady process of deterioration of the vitamin A over time in the extruded rice. At higher temperatures and humidity, this process was accelerated, with 22%–35% of the vitamin A lost after three months. Although for extruded rice, overages could be increased by a third, to counteract this loss of vitamin A during storage, our earlier paper on losses during cooking showed an additional high loss of vitamin A during the cooking process [10], with up to 80% of the vitamin A lost. Therefore, it is questionable whether rice is a suitable food vehicle for fortification with vitamin A. Other food items, such as vegetable oil might be more efficient to increase the overall vitamin A status of a population [16] although the quality of the vegetable oil determines the stability of vitamin A [17].

To prevent iron deficiency, fortifying all rice consumed in Cambodia with iron at these calculated levels would substantially increase iron intake. However, currently it is less clear whether this would lead to a significant increase in iron status of the population. Two recent studies conducted in Cambodia showed a low prevalence of iron deficiency in women of reproductive age [18] and in school children [19]. Moreover, mixed results have been reported on the effectiveness of iron-fortified rice on improving iron status. Extruded rice with only iron increased iron status in Indian school children [20], but rice fortified with zinc, iron and vitamin A had no impact on iron status in Cambodian [21] or Thai school children [22]. Also, in another Indian study, a higher dose of iron (12.5 mg) in fortified rice, combined with several other micronutrients including zinc, failed to improve iron status also [23]. Perhaps most worrying, in a Cambodian trial with fortified rice including iron, the prevalence of hookworm infection was significantly increased in school children receiving fortified rice [24]. In addition, since 2014, over 40 producers of fish and soy sauce are fortifying their products with iron EDTA (Ethylenediaminetetraacetic acid iron). Since the start of the program, almost 6.4 million litres of fish and soy sauce have been fortified. With a daily consumption of 10 mL and an average fortification level of 323 ± 146 mg/L, fortified sauces increase daily iron intake by 3.3 ± 1.5 mg among consumers (personal communication, A. Laillou). This intervention has proven to be effective in Vietnam [6] and Cambodia [21,23].

Zinc deficiency is considered an urgent public health problem in Cambodia, given the high levels of stunting (>30% of the children <5 years of age were stunted in 2014). Also, the prevalence of zinc deficiency in Cambodian school children was high, with >80% of children participating in a rice fortification study having low plasma zinc concentrations [25]. Therefore, fortification of rice with zinc could be an effective solution to improve the zinc status of the Cambodian population.

## 5. Conclusions

The results of this study show that losses of iron and zinc in fortified rice during storage are negligible, even over longer periods of time, and at high temperature and humidity. Therefore addition of zinc and iron to rice could be an effective strategy to improve zinc and iron status of the Cambodian population. Before being able to make a solid recommendation on the benefits of iron-fortified rice for Cambodia, more updated information on (i) the national prevalence of iron deficiency; (ii) the etiology of anemia in Cambodia and (iii) the reach of other iron deficiency control strategies are needed.

In contrast, losses of vitamin A were considerable, especially at high temperature and humidity, with over 80% of the vitamin A lost after three months in coated fortified rice. The current coating techniques for making rice premix appear to be inadequate to guarantee vitamin A concentrations, and adding vitamin A to the premix for coated fortified rice is therefore not recommended. Vitamin A was more stable in extruded rice, although after one year at high temperature and humidity 50%–60% of the vitamin A was lost. The addition of vitamin A in rice is questionable due to the excessive amount of overage needed to overcome the losses during storage and cooking.
